# Efficient expression and purification of rat CRP in *Pichia pastoris*

**DOI:** 10.3389/fimmu.2024.1465365

**Published:** 2024-08-26

**Authors:** Bin Cheng, Yu-Long Tang, Ya-Fei Gou, Jing-Yi Li, Tian-Hao Xu, Li Zhu

**Affiliations:** ^1^ Key Laboratory of Preclinical Study for New Drugs of Gansu Province, School of Basic Medical Sciences, Research Unit of Peptide Science, Chinese Academy of Medical Sciences 2019RU066, Lanzhou University, Lanzhou, China; ^2^ Ministry of Education (MOE) Key Laboratory of Cell Activities and Stress Adaptations, School of Life Sciences, Lanzhou University, Lanzhou, China

**Keywords:** C-reactive protein, rat CRP, protein expression, purification, *Escherichia coli*, *Pichi pastoris*

## Abstract

C-reactive protein (CRP) plays a crucial role in the diagnosis and monitoring of the non-specific acute phase response in humans. In contrast, rat CRP (rCRP) is an atypical acute-phase protein that possesses unique features, such as a possible incapacity to trigger the complement system and markedly elevated baseline plasma concentrations. To facilitate *in vitro* studies on these unique characteristics, obtaining high-quality pure rCRP is essential. Here we explored various strategies for rCRP purification, including direct isolation from rat plasma and recombinant expression in both prokaryotic and eukaryotic systems. Our study optimized the recombinant expression system to enhance the secretion and purification efficiency of rCRP. Compared to traditional purification methods, we present a streamlined and effective approach for the expression and purification of rCRP in the *Pichia pastoris* system. This refined methodology offers significant improvements in the efficiency and effectiveness of rCRP purification, thereby facilitating further structural and functional studies on rCRP.

## Introduction

C-reactive protein (CRP) is a widely recognized biomarker of inflammation, commonly utilized as a representative acute phase reactant in clinical settings (1, 2). This pentraxin is identified in most vertebrates, such as mice, rats, and humans, and in invertebrates like the horseshoe crab (*Limulus polyphemus*) (3–6). CRP is primarily synthesized by the liver and possesses a diameter exceeding 10 nm (7–9). In response to infection or tissue injury, human blood concentrations of CRP can surge from under 1 μg/mL to more than 500 μg/mL within a 24-hour period. Multiple clinical studies indicate that CRP functions as both a non-specific marker of inflammation associated with cardiovascular diseases and a predictor of future acute cardiovascular events, potentially influencing the development of atherosclerosis (1, 10–15).

In contrast to humans, rats exhibit significantly higher basal plasma CRP concentrations, ranging from 300-500 μg/mL, which is approximately 100 times greater than that found in humans, and comparable to levels seen during the acute phase (16, 17). However, in response to inflammatory stimuli, rat serum CRP levels only increase by 2-3 times during the acute phase, indicating that CRP in rats does not conform to the typical acute-phase protein profile (9, 18, 19). Despite sharing 70% amino acid homology (20), rat CRP (rCRP) has been reported to be incapable of activating complement, unlike human CRP (21–23). Activation of complement by rCRP has only been investigated with the C-polysaccharide of *Streptococcus pneumoniae* (24). However, assays have been developed for complexes of CRP and activated complement fragments, demonstrating that these complexes accurately represent CRP-induced complement activation (25). It has been shown through research that rCRP is capable of activating autologous complement, and the activation of complement when binding to a suitable ligand is an important biological function of this pentraxin (26).

Research demonstrates that CRP is primarily produced by the liver and circulates in the bloodstream (27). Historically, the purification of CRP from acute-phase sera and other protein expression systems has predominantly relied on affinity column chromatography. This process entails the utilization of Sepharose 4B substituted with p-Aminophenyl Phosphoryl Choline, a ligand designed to selectively bind to CRP (28–30). However, this method is expensive and time-consuming. Additionally, serum amyloid P (SAP), another acute-phase reactant found in serum, binds directly to the galactose residues of Sepharose 4B, which is a major source of contamination in CRP purification (30, 31). The intricate nature of blood components, coupled with the presence of SAP, increases the difficulty of CRP purification from blood. Although various expression systems have been established for recombinant human and mouse CRP production (30, 32, 33), there have been no investigations on rCRP purification. Unicellular microorganisms are commonly favored as host systems for their rapid generation times, abundant biomass production, and well-established methods for manipulation and modification (34–38). While bacterial heterologous protein expression systems can produce large quantities of protein, but often face signification limitations regarding protein solubility, proper folding, and post-translational modifications, particularly disulfide bonds which are often crucial for correct protein folding (9, 34). Although yeast expression systems typically yield lower quantities, they offer an environment that closely mimics the eukaryotic cell for the emerging protein, incorporating similar chaperone proteins, a non-reducing cytoplasm, and secretion mechanisms (39).

*Pichi pastoris* is a well-known eukaryotic model organism used for the production of heterologous proteins (36, 38). Unlike prokaryotic expression system, *P. pastoris* has the capacity for post-translational modifications and secretion, significantly reducing the cost of post-fermentation *in vitro* purification and modification (38). Additionally, *P. pastoris* exhibits increased resistance to low pH levels, elevated sugar and ethanol concentrations, and high osmotic pressure, rendering it well-suited for use in industrial fermentation processes (39). In this study, we isolated and purified rCRP from various sources, including blood components, prokaryotic, and eukaryotic expression systems. We then conducted a comparative analysis, revealing that utilizing a *P. pastoris* expression system enables the efficient production of abundant rCRP with preserved structure and functionality. This method offers a rapid, straightforward, and effective approach to rCRP purification. 

## Materials and methods

### Purification of rCRP from blood

The protocol utilized in this study was approved by the Ethics Committee of Lanzhou University and complies with the Guide for the Care and Use of Laboratory Animals, as outlined by the USA National Institute of Health. Male Wistar rats, weighing 280-300 g were utilized for the experiment. The animals were housed individually in metabolic cages and maintained under a 12-hour light/12-hour dark cycle with constant humidity. They had ad libitum access to commercial rat chow and tap water. Prior to the experiments, the rats were fasted overnight.

To prepare rat serum CRP, each clinically healthy rat received a subcutaneous injection of 0.5 mL turpentine oil. After 48 hours, blood was collected, and serum was obtained by centrifugation of the clotted blood at 3,000 rpm for 30 minutes, followed by centrifugation of the supernatant at 14,000 rpm for 30 minutes. The serum was supplemented with 1 mM PMSF before homogenization and filtered through a 0.22 μm filter membrane. The serum was then diluted six-fold with a buffer containing 20 mM Tris-HCl (pH 7.5), 150 mM NaCl, 2 mM CaCl_2_, and 0.02% NaN_3_. The diluted serum was applied to Pierce™ p-Aminophenyl Phosphoryl Choline Agarose resin (Thermo Scientific) and incubated at 4°C for 1 hour. Proteins were eluted using the binding buffer supplemented with 10 mM Phosphorylcholine Chloride Calcium Salt Tetrahydrate (TCI, Tokyo Chemical Industry). The eluted protein was concentrated using a 30-kDa cut-off Centricon (Millipore) and further purified via size exclusion chromatography (SEC) (Superdex-G200 Increase column, GE Healthcare) in a buffer containing 20 mM Tris (pH 7.5), 150 mM NaCl, 2 mM CaCl_2_, and 0.02% NaN_3_. The peak fractions corresponding to the rCRP were pooled and concentrated.

### Plasmid construction for vector construction, transformation and expression in *Escherichia coli*

The total RNA was extracted using RNAiso Plus reagent (Takara, Shiga, Japan; catalog number: 9109; lot number: AKA3402, AKA5802). The cDNA was synthesized from 2 μg of total RNA using PrimeScript RT Master Mix system (Takara; catalog number: RR036A; lot number: AK4102, AK4403). The rat nucleotide sequence encoding CRP was obtained from NCBI (NM_017096.3). The DNA was amplified using polymerase chain reaction (PCR) with PrimeSTAR HS DNA Polymerase (Takara; catalog number: R010A; lot number: N5301DA) and specific primer sequences (forward: 5′-CGG GGT ACC CAT GAA GAC ATG TCT AA-3′; reverse: 5′-CCG GAA TTC AGG ACT CAC AAC AGT C-3′).

The PCR product comprising the coding sequences of wild-type rCRP was subcloned into pET42C (Novagen) plasmids between the *NdeI* and *EcoRI* sites. This was carried out in-frame with Strep-tag^®^II (WSHPQFEKGGGSGGGSGGSAWSHPQFEK), which was fused at the N-terminus of the full-length rCRP using the T4 DNA Ligation Kit Ver.2.1 (Takara; Code number: 6022). The *E. coli* DH5α competent cells were employed for the amplification and cloning of plasmids. When required, the N-terminus of CRP was fused with the signal peptide of alkaline phosphatase (ALP) to facilitate secretion. *E. coli* BL21 (DE3) competent cells were transformed with 50 ng of the constructed plasmid rCRPwt-pET42c and plated on LB agar (1% Tryptone, 0.5% Yeast Extract, 1% NaCl, 1.5% agar, 50 μg/mL kanamycin, pH 7.5). After overnight incubation at 37°C, colonies were obtained from the transformation plates, which were subsequently validated using colony PCR and sanger sequencing. The chosen cell strain was cultured in 1 mL of LB medium, incubated at 37°C with agitation at 220 rpm for 3.5 hours, and then diluted into 1 L of fresh medium. The cells were evenly distributed into 2.8-L baffled flasks and cultivated at 37°C with shaking until the optical density at 600 nm (OD_600_) reached 0.6, at which point the temperature was reduced to 16°C. After stabilization, isopropyl β-D-1-thiogalactopyranoside (IPTG) was introduced at a final concentration of 500 μM, and expression was sustained for 24 hours with shaking at 220 rpm. After centrifuging the cells at 5000 × *g* for 10 minutes, and the supernatants was collected as conditioned media.

To purify the protein, 1 mM PMSF was added to the conditioned media before homogenization. The media was then concentrated using VIVAFLOW 200 30,000 MWCO HY (Sartorius, UK; Catalog number: VF20H2; Lot number: 184800967; 191900967). After ultra-centrifugation at 20,000 × *g* for 45 minutes, the supernatant was collected and applied to the Strep-tag^®^ II Fusion Tag affinity gel (Sigma) for 1 hour incubation at 4°C. The resin underwent four rinses, each using 5 mL of buffer consisting of 20 mM HEPES, pH 7.5, 150 mM NaCl, 2 mM CaCl_2_, and 0.02% NaN_3_. Following each rinse, the resin was thoroughly washed with the same buffer before being eluted with the addition of 5 mM D-Desthiobiotin (Sigma). Following the elution from the Strep-tag^®^ II Fusion Tag affinity gel column, the sample was loaded onto the Pierce™ p-Aminophenyl Phosphoryl Choline Agarose resin and incubated at 4°C for 1 hour. The proteins were then eluted by wash buffer supplemented with 10 mM Phosphorylcholine Chloride Calcium Salt Tetrahydrate. The protein eluent underwent concentration through a 30-kDa cut-off Centricon and was further purified using a Superdex-G200 Increase column (GE Healthcare) in a buffer solution consisting of 20 mM HEPES, pH 7.5, 150 mM NaCl, 2 mM CaCl_2_, and 0.02% NaN_3_. The peak fractions that matched rCRP were collected and concentrated.

### *P. pastoris* vector construction, transformation and expression

The plasmid rCRPwt-pPIC9K, encompassing the nucleotide sequence encoding rCRP, was amplified by PCR using PrimeSTAR HS DNA Polymerase (Takara; catalog number: R010A; lot number: N5301DA) with the following primers: 5′-CCG GAA TTC CAT GAA GAC ATG TCT AAA CAG GCC TTC GTA TTT CCC GGA GTG TCA GCT ACT G-3′ and 5′-ATA GTT TAG CGG CCG CTT AAT GAT GAT GAT GAT GAT GGG CGC CGG ACT CAC AAC AGT CAG -3′. This was done to incorporate *EcoRI* and *NotⅠ* restriction sites and the Strep-tag^®^II substituted with a hexa-histidine coding region fusion at the C-terminus. The fusion protein was produced in the *P. pastoris* GS115 wild type strain through the utilization of methanol-inducible pPIC9K yeast expression vectors (Invitrogen, USA). The pPIC9K vector contains a *Saccharomyces cerevisiae* α-factor secretion signal sequence at the beginning of the insert protein gene and the alcohol oxidase gene (AOX1) promoter, which initiates the expression of the recombinant protein in the presence of methanol as the carbon source. The accuracy of the nucleotide sequence was confirmed through DNA sequencing.

The plasmid constructs were linearized with *SalI* (Takara; Code No.1080AH) before being converted into *P. pastoris*, in order to cleave the HIS4 coding region for integration into the HIS4 locus of the pPIC9K plasmid (His^-^). The electroporation method was used to integrate plasmid into its genome in a stable fashion using the Pulse cell transfection system (GEMINI X2, BTX). Cells were pulsed in 0.2 cm sterile electroporation cuvettes at 1.5 kV, 25 μF, 400 Ω. Following pulsing, 1 mL of cold 1 M sorbitol was promptly added to the cuvette. The cells were subsequently cultured on minimal dextrose (MD) agar plates (1.34% yeast nitrogen base (YNB), 4×10^-5^% biotin, 2% dextrose, 1.5% agar) for the purpose of selecting His^+^ transformants. Colonies exhibiting the His^+^ phenotype resulting from the transformation were isolated and transferred to YPD agar plates (1% yeast extract, 2% peptone, 2% dextrose, 2% agar) with varying concentrations of G418 (ranging from 0 to 8 mg/mL). After a 2-day incubation period, the growth response to different G418 concentrations was assessed. Subsequently, colonies demonstrating successful integration of the expression vectors into their genomes were identified. The genotypes of the His^+^ transformants were subsequently validated through PCR analysis.

Single colonies of the recombinant *P. pastoris* were inoculated into 25 mL YPD (1% yeast extract, 2% peptone, and 2% dextrose) and incubated at 30°C with shaking at 220 rpm for 18 hours until the culture reached an OD_600_ of 1.3-1.5. Next, the cells are transferred to a 1 L flask containing buffered minimal glycerol medium (BMGY - containing 100 mM potassium phosphate, pH 6.0, 1.34% YNB, 4×10^-5^% biotin, 1% glycerol, 2% peptone, and 1% yeast extract) and grown overnight at 28°C with shaking at 220 rpm until the culture reaches an OD_600_ of 2-6, which typically takes around 18-24 hours. The cells will be in log-phase growth as they were harvested from BMGY and re-suspended in 1 L of buffered minimal methanol medium (BMMY -Replacing 1% glycerol in BMGY with 1% methanol) to induce protein expression. The flask was covered with 2 layers of sterile gauze and return to the incubator to continue growing at 30°C with constant shaking at 220 rpm for 5 days.

To maintain induction, 100% methanol was added to a final concentration of 1% methanol every 24 hours. The culture medium was then separated from the cells by centrifugation at 5000 × *g* for 45 minutes at 4°C. After collecting the supernatant, it was supplemented with 1 mM PMSF before homogenization. Using VIVAFLOW 200 30,000 MWCO HY (Sartorius, UK; Catalog number: VF20H2; Lot number: 184800967; 191900967) for concentration, then the supernatant was ultra-centrifuged at 20,000 × *g* for 45 minutes to collect the centrifugal supernatant. Nickel Sepharose beads (GE Healthcare, Sweden) were used for the batch binding purification of His-tagged rCRP, following the manufacturer’s protocol. The collected medium was added to column of Ni^2+^-NTA resin and incubated at 4°C for 1 hour. The column was initially rinsed with a buffer solution (20 mM Tris–HCl (pH 7.5), 150 mM NaCl, 2 mM CaCl_2_). Subsequently, elution was performed using a solution containing 500 mM imidazole in the buffer, and the eluate fractions were then collected and stored at 4°C. Analysis of the elution fractions was conducted using sodium dodecyl sulfate-polyacrylamide gel electrophoresis (SDS-PAGE).

In the preceding stage, fractions containing rCRP were combined and further purified using Pierce™ p-Aminophenyl Phosphoryl Choline Agarose resin. Rat CRP sample was loaded onto a column, washed with a buffer solution, and then eluted with an additional 10 mM of Phosphorylcholine Chloride Calcium Salt Tetrahydrate. The resulting eluate fractions were collected, stored at 4°C, and subsequently analyzed using SDS-PAGE. The proteins were dialyzed in a buffer solution (20 mM Tris–HCl (pH 7.5), 150 mM NaCl, 2 mM CaCl_2_, 0.02% NaN_3_) for 48 hours, with the dialysate being refreshed every 12 hours. After dialysis, the proteins were concentrated, their concentration was measured, and they were subsequently packaged and promptly frozen in liquid nitrogen for storage at -80°C.

### Protein assay and gel electrophoresis

Protein concentrations were determined using Bradford assay (Bio-Rad) method using a commercial kit (Pierce™ BCA Pritein Assay Reagent A, Thermo scientific, REF number: 23228; Lot number: SK255590; Pierce™ BCA Pritein Assay Reagent B, Thermo scientific, Prod number: 1859078; Lot number: QK226547), using bovine serum albumin as the standard protein. The purity of rCRP was assessed by 12% [w/v] SDS-PAGE and 1/20 SDS-PAGE followed by Coomassie Blue and silver staining, according to previously described methods (40, 41). The SDS-PAGE gel image was scanned using a Canon 9000F Mark II scanner at 8-bit grayscale resolution.

### Western blotting

Samples were separated on 12% [w/v] SDS-PAGE gels. The blots were blocked for 1 hour in TBS-T (50 mM Tris-HCl pH 7.4, 150 mM NaCl, and 0.1% [v/v] Tween 20) with 5% [w/v] fat-free milk, incubated overnight at 4˚C with specific antibodies. Detection of extracellular and intracellular soluble proteins secreted by *E. coli* expressing rCRP was achieved through the use of an anti-strep mAb (at a dilution of 1:30,000; Sangon Biotech, Shanghai, China; catalog number: D191106) in conjunction with HRP-labeled goat-anti-mouse IgG (at a dilution of 1:40,000). The rat CRP secreted from yeast was detected using the primary antibody His-Tag Antibody (Proteintech, USA; Catalog number: 66005-I-Ig; Lot number: 10004365) in conjunction with the secondary antibody, horseradish peroxidase (HRP)-conjugated goat anti-mouse IgG antibodies (dilution 1:30,000; Gibco BRL). The membranes were blocked with 5% non-fat milk and then visualized using ECL.

### Enzyme-linked immunosorbent assay

ELISAs were conducted following standard procedures with slight adjustments (9, 31, 42). Specifically, 10 μg/mL of phosphorylcholine keyhole limpet hemocyanin (PC-KLH) (Santa Cruz Biotechnology, USA; Catalog number: sc-396,490; Lot number: J0915) was immobilized onto microtiter plate (Thermo Fisher Scientific) in TBS-Ca (10 mM Tris–HCl, pH 7.5, 140 mM NaCl, and 2 mM CaCl_2_) for 12 hours at 4°C. All subsequent steps were performed at 37°C, including washing the wells with TBS-Ca solution containing 0.02% Nonidet P-40, and blocking with 1% BSA/TBS-Ca for 1 hour. The supernatant was centrifuged at 5000 × g to eliminate cells, then diluted two-fold with TBS buffer and incubated in 96-well plates for one hour. Binding detection was achieved using His-Tag Antibody (Proteintech, USA; Catalog number: 66005-I-Ig; Lot number: 10004365) along with horseradish peroxidase (HRP)-conjugated goat anti-mouse IgG antibodies (1:30,000; Gibco BRL). The reaction was initiated by adding TMB (Sigma-Aldrich; Catalog number: T2885; Lot number: WXBC2414V) to the wells and terminated with 1mM H_2_SO_4_. Absorbance readings were subsequently taken at 450 nm and 570 nm.

### Mass spectrometry analysis

The corresponding stripe on the SDS-PAGE gel was subjected to in-gel digestion with Trypsin Gold (Promega) protease after washing. The peptides were resuspended in 0.1% formic acid (FA) and subsequently subjected to analysis using an Orbitrap Fusion Lumos mass spectrometer (Thermo Fisher Scientific) coupled online to an EASY-nLC 1200 system, following established protocols (31). Upon desalting with a Zip-tip C18 (Millipore), the tryptic peptides were eluted with 0.5 ml of matrix solution (a-cyano-4-hydroxycinnamic acid, 5 mg/ml in 50% acetonitrile, 0.1% trifluoroacetic acid, 25 mM ammonium bicarbonate). The peptide mass and fragmentation spectra resulting from the experiment were analyzed using Proteome Discoverer 2.3 to search for matches within the protein sequences of rCRP sourced from the NCBI database.

### Electron microscopy

Negative staining was performed according to previously described methods (43–46). In brief, CRP was diluted to 0.15-0.3 mg/mL in 20 mM Tris (pH 6.8), 150 mM NaCl, 2 mM CaCl_2_, and incubated for 1-2 hours at 18°C before staining. After adding 4 μL droplet of the sample to a freshly glow-discharged carbon-coated 300 mesh copper EM grid for 10 seconds, the grid was then stained with 1% uranyl acetate for 30 seconds. The sample was observed with an FEI Talos electron microscope operating at 200 kV. Micrographs were recorded using Ceta-II CMOS 4K×4K camera at a nominal magnification of 120,000 × with a pixel size of 1.25 Å.

## Results and discussion

### Purification of rCRP from serum

**Figure 1:**
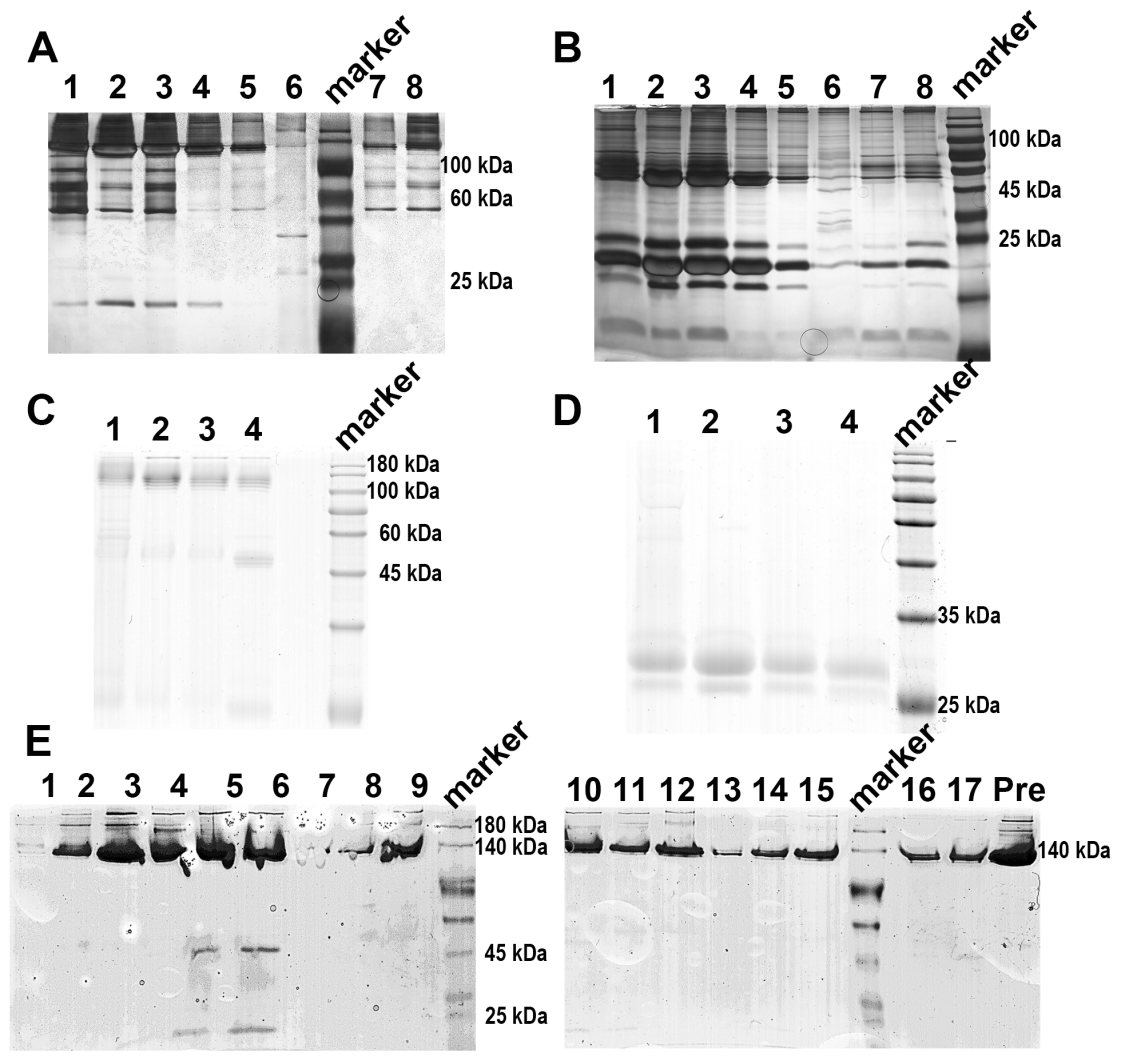
SDS-PAGE analysis of rCRP during downstream purification from rat serum. **(A)** 1/20 SDS-PAGE analysis of PCh-column with 10 mM PCh buffer elution; Lane 1-6, eluted fraction samples of 10 mM PCh buffer; Molecular weight marker proteins from top to bottom: 180 kDa, 140 kDa, 100 kDa, 75 kDa, 60 kDa, 45 kDa, 35 kDa, 25 kDa, 15 kDa, 10 kDa; Lane 7-8 eluted fraction samples of 5 mM EDTA buffer. **(B)** Perform SDS-PAGE analysis of PCh-column with 10 mM PCh buffer elution, boil the sample in a water bath for a period of 10 minutes, in the same order as shown in **(A)**. The presence of numerous miscellaneous proteins in **(A, B)** was attributed to cell hemolysis during processing, prompting efforts to minimize hemolysis during CRP purification from blood. **(C)** 1/20 SDS-PAGE analysis of PCh-column with 10 mM PCh buffer elution; Lane 1-4, eluted fraction samples of 10 mM PCh buffer; **(D)** SDS-PAGE analysis of PCh-column with 10 mM PCh buffer elution, in the same order as shown in **(C)**; **(E)** 1/20 SDS-PAGE analysis of rCRP eluted fraction from DEAE chromatography; Fraction samples: 1-17; Pre, pre-sample before column.

**Figure 2:**
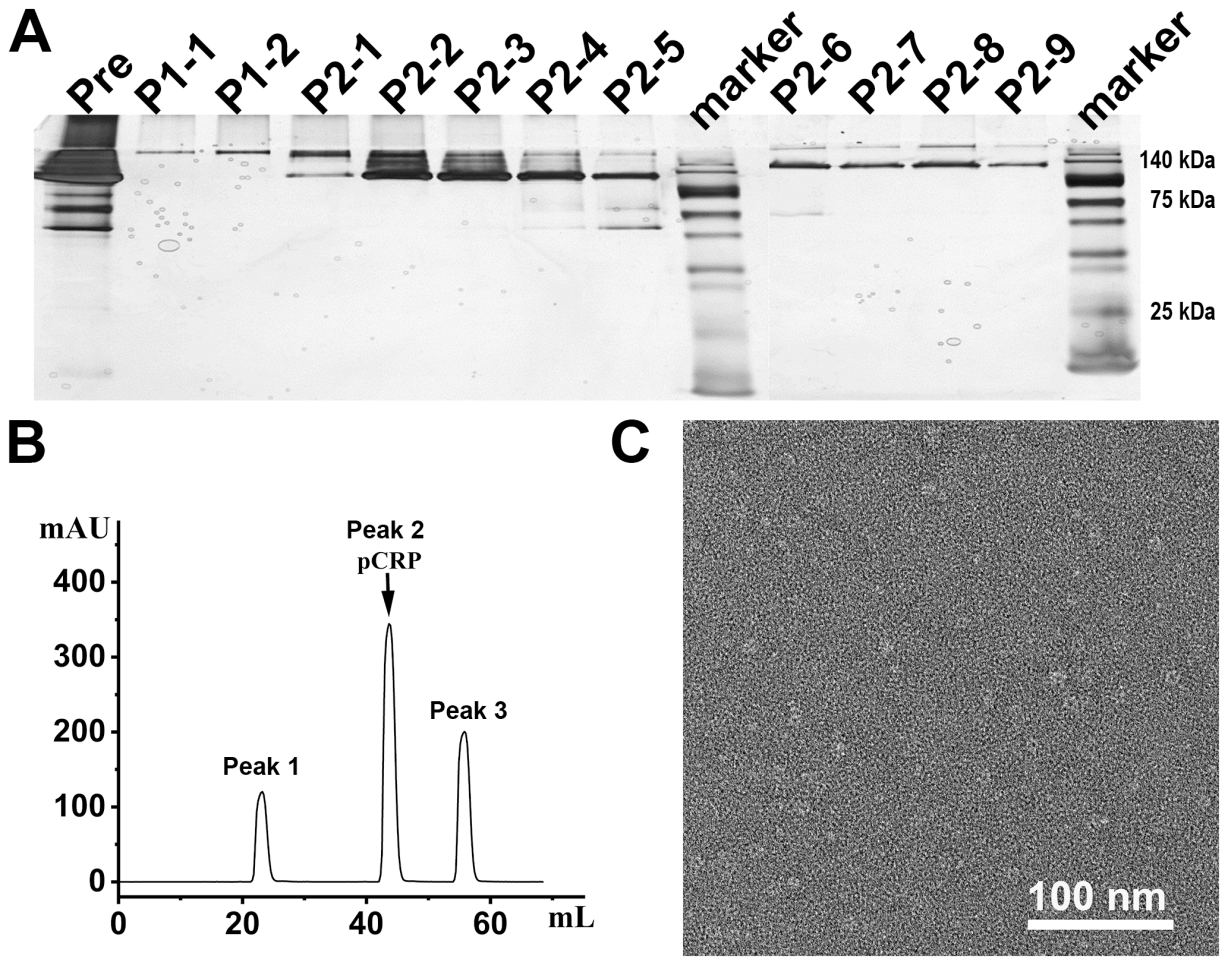
The SEC assay and electron microscopy for negative staining analysis of rCRP during downstream purification from serum. **(A)** 1/20 SDS-PAGE analysis of SEC purification sample; Pre, pre-sample before column; P1-1 and P1-2 represent the results for peak 1 detection, while P2-1 to P2-9 correspond to the results for peak 2 detection; All samples run under non-reducing conditions. **(B)** The SEC assay indicated that the rCRP with an intact pentameric structure was observed at peak 2. **(C)** Negative staining of rCRP purified from serum.

When applying 1/20 SDS-PAGE and SDS-PAGE analysis to rCRP purified from serum using Pierce™ p-Aminophenyl Phosphoryl Choline Agarose resin, there is a significant enrichment of rCRP upon elution with 10 mM PCh-buffer (**Figures 1A, B**). A significant amount of heterologous proteins is inevitably introduced as a result of hemolysis during blood collection and processing in rats. Consequently, rigorous protocols were established for subsequent experiments to mitigate cell hemolysis. By preventing cell hemolysis, the presence of extraneous proteins in the serum was significantly reduced, leading to a more purified rCRP (**Figures 1C, D**). Anion exchange chromatography was then employed, allowing the target protein to bind to DEAE at pH 6.0 and be eluted with a gradual increase in salt concentration. High and low molecular weight impurities were successfully removed after the target protein (**Figure 1E**), although some high molecular weight impurities remained difficult to eliminate. Following DEAE chromatography, the eluted sample underwent concentration and buffer exchange through SEC. The purified protein was observed as a transparent, colorless solution with a neutral pH. Additionally, analysis by 1/20 SDS-PAGE revealed a prominent band near the anticipated molecular weight of approximately 120 kDa (**Figures 2A, B**). The electron microscopy analysis of rCRP revealed the presence of a pentamer ring when utilizing negative staining (**Figure 2C**).

### Expression and purification of rCRP in a prokaryotic expression system

**Figure 3:**
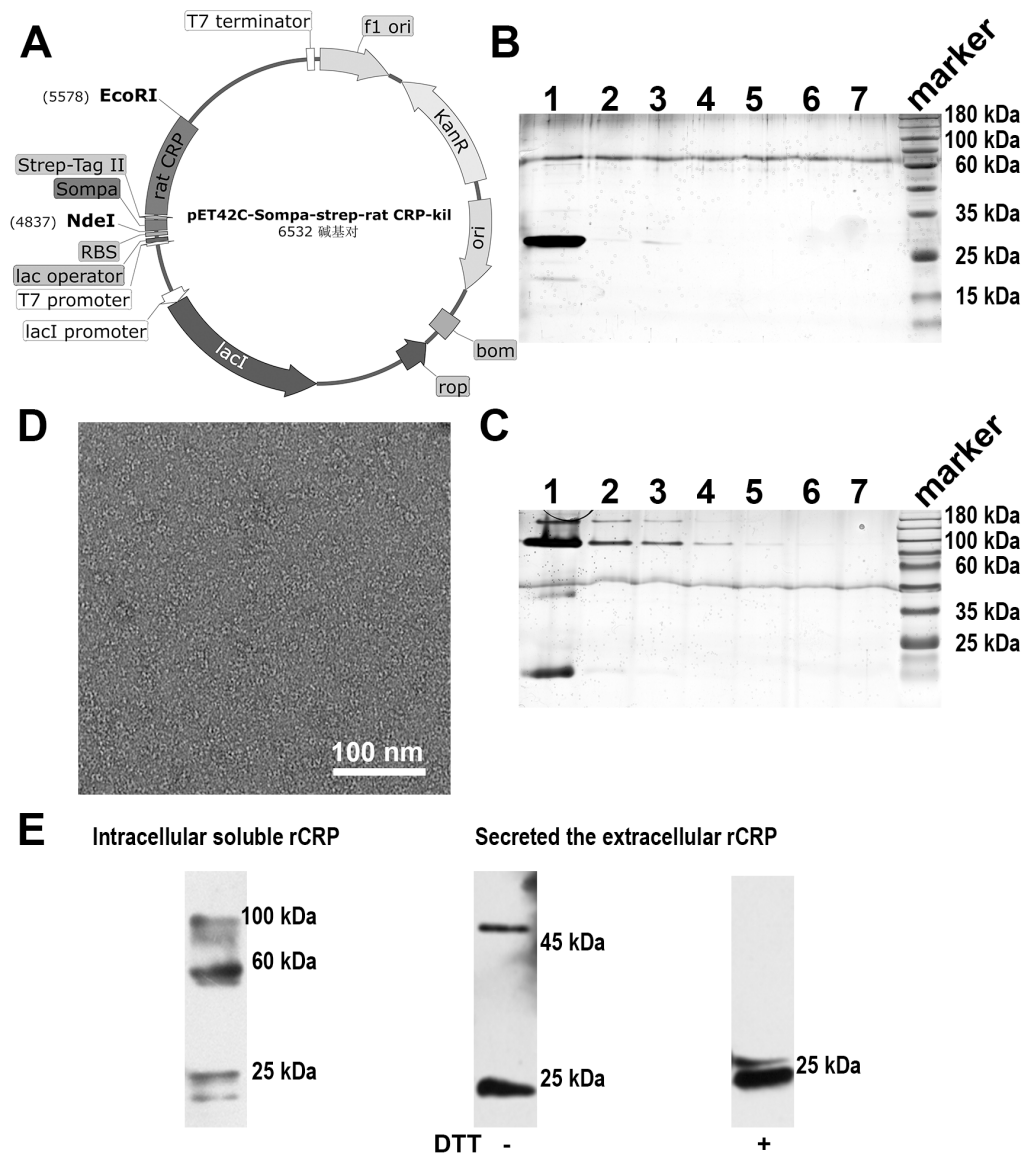
Rat CRP expression and purification in **(*E*)** *coli* strains BL21 (DE3) system. **(A)** Genetic maps of pET42C-Sompa-strep-rCRP-kil plasmid, with a Strep-tag^®^II fusion tag constructed at the N-terminus, generated from SnapGene software. **(B)** SDS-PAGE analysis of non-reduced samples of prokaryotic expression rCRP, which extracts from medium through the in tandem purification of Strep-Tag^®^ II Fusion Tag affinity gel column and Pierce™ p-Aminophenyl Phosphoryl Choline Agarose resin. Lane 1-7, fraction sample of affinity column. **(C)** 1/20 SDS-PAGE analysis of non-reduced samples, the order of sample same as **(B)**; Some trace higher molecular weight impurities in lanes. **(D)** Negative staining of rCRP expressed in *E. coli*. **(E)** The expression of rCRP in *E. coli* was examined using Western blot analysis, focusing on both intracellular soluble proteins and secreted proteins in their oxidized and reduced states.

The integration of rCRP gene cassettes into plasmids within the pET42C expression vector (**Figure 3A**). Following its transformation into the *E. coli* strain BL21(DE3), the vector facilitated the production of the rCRP protein. Subsequent purification steps involving a Strep-Tag^®^ II Fusion Tag affinity gel column and Pierce™ p-Aminophenyl Phosphoryl Choline Agarose Fast Flow columns resulted in a reduced protein yield. Moreover, according to SDS-PAGE and 1/20 SDS-PAGE analysis, the protein band was not singular and contained many impurities (**Figures 3B, C**). Electron microscopy revealed the presence of numerous non-pentameric impurities in proteins during negative staining (**Figure 3D**). The diminished secretion capacity observed in host cells carrying the rCRP gene may be attributed to the structural composition of CRP, characterized by four cysteine residues per molecule, coupled with constraints within the cellular secretory pathways (16, 28, 47). Major bottlenecks in the secretion of recombinant proteins from *E. coli* bacterial cells include membrane translocation, signal peptide recognition, protein folding, disulfide bond formation, and posttranslational processing (30). Despite originating from a prokaryotic expression system, rCRP maintained its native pentameric structure (**Figure 3D**). Immunoblotting analysis showed that expression in *E. coli* cells there are many forms of CRP in rats, may be the result of no modification after translation different folding state (**Figure 3E**). While the secreted protein fraction maintains the correct pentamer conformation, its expression level is unfortunately low.

### Expression and purification of rCRP in a yeast expression system

**Figure 4:**
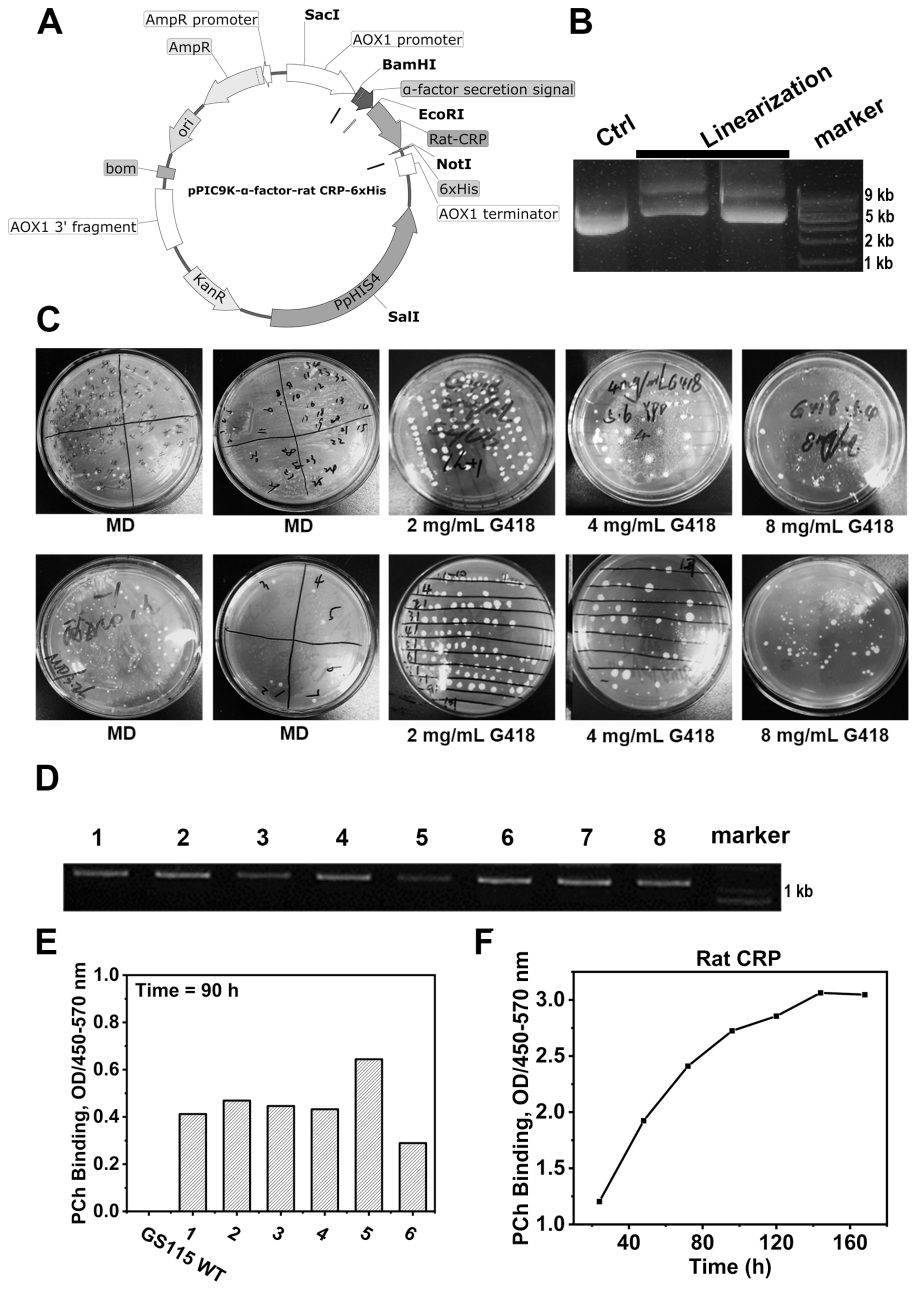
Rat CRP expression in *P. pastoris* strains GS115 system. **(A)** Genetic maps of pPIC9K-α-factor-rCRP-6×His plasmid with a 6×His tag fused at the C-terminus, generated from SnapGene software. **(B)** Linearization of plasmids; Lane 1, unlinearized ring plasmid as contral; Lane 2-3, the linearized plasmid was cleaved by restriction enzyme *Sal1*; Lane 4, molecular weight marker DNA. **(C)** MD agar plates for the selection of His^+^ transformants and different concentration of G418 filter multiple copies. **(D)** The genotypes of multiple copies transformants were verified by PCR. **(E)** Screening of yeast strains overexpressing rCRP. After inducing expression for 90 hours with 1% methanol, the supernatant was collected by centrifugation. ELISA was performed to assess the binding between rCRP, secreted into the culture medium, and its pattern recognition ligand PCh. The aim is to identify high-expression strains by screening from high-copy strains. **(F)** Protein expression profile of rCRP from the identified high-expressing yeast strains. The high-expressing bacterial strains were induced for expression, with 1 ml samples taken every 24 hours for 7 consecutive times. The supernatant was separated by centrifugation to measure the expression level of rat CRP. At 140 hours, protein levels reach their peak, but subsequently plateau as a result of nutrient depletion and waste buildup.

The construction of plasmids containing rCRP gene cassettes was accomplished using the pPIC9K expression vector (**Figure 4A**), with the cleavage results of the modified plasmid using the *SalⅠ* restriction enzyme (**Figure 4B**). The recombinant plasmid was effectively transformed into yeast cells to facilitate the production of recombinant rCRP within the methylotrophic yeast expression system (**Figure 4C**). After two rounds of screening using Minimal Dextrose (MD) agar plates to isolate His^+^ transformants and various G418 concentrations to filter multiple copies, we randomly selected eight distinct *P. pastoris* clones. The clones were identified using PCR (**Figure 4D**) and sanger sequencing. Subsequently, those clones with accurate sequences were chosen for screening high-expression strains (**Figure 4E**). The strain demonstrating the highest expression level was chosen for real-time expression monitoring (**Figure 4F**) and subsequently employed as the host for protein expression and purification.

**Figure 5:**
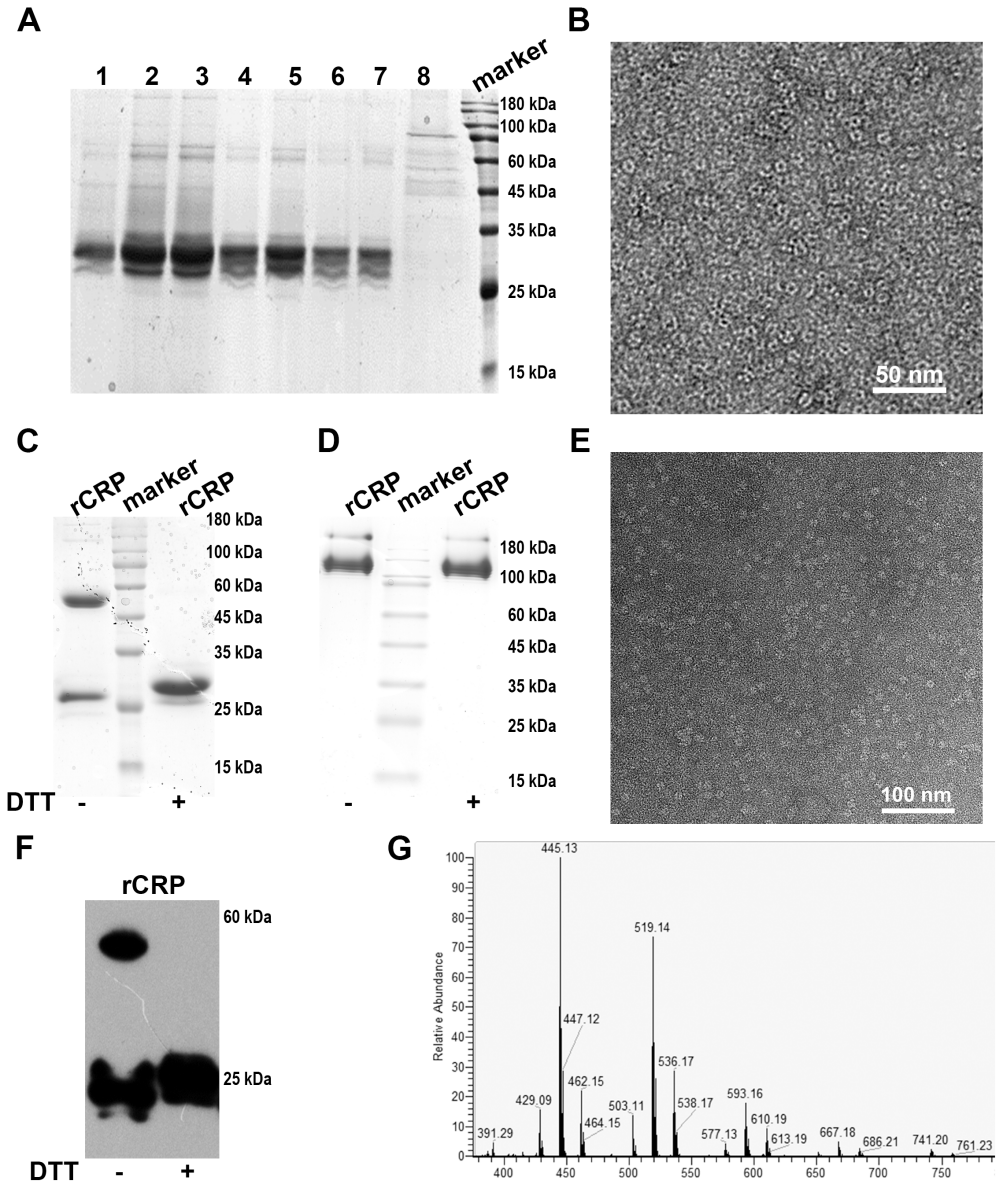
Analyses of rCRP obtained after the tandem purification using a nickel sepharose column and PCh-column. **(A)** SDS-PAGE of Ni^2+^-NTA affinity chromatography elution; Lane 1-7, fraction samples of nickel column eluted with 500 mM imidazole; Lane 8, flow-through samples from nickel column. **(B)** Negative staining of rCRP obtained from Ni^2+^-NTA affinity chromatography. **(C)** SDS-PAGE analysis rCRP, which obtained from Ni^2+^-NTA affinity chromatography were concentrated with a 30 kDa cut-off Centricon and purified further with PCh column; Fraction samples of PCh-column eluted with 10 mM PCh; With non-reduced/reduced samples, right lane added 20 mM Dithiothreitol (DTT). **(D)** 1/20 SDS-PAGE analysis of rCRP with non-reduced/reduced samples, orders same as **(C)**; **(E)** Negative staining of rCRP obtained from Ni^2+^-NTA affinity chromatography and PCh affinity chromatography in tandem. **(F)** The expression of recombinant rCRP secreted by yeast was examined using Western blot analysis. As observed in previous SDS-PAGE results, the dimeric structure of rCRP was eliminated with DTT added, resulting in the presence of only the monomeric form. **(G)** Mass spectrometry analysis of purified rat CRP.

The purification of secreted rCRP protein from culture broth was achieved using Ni^2+^-NTA and Pierce™ p-Aminophenyl Phosphoryl Choline Agarose Fast Flow column affinity chromatography with a linear gradient elution procedure. The affinity chromatogram of secreted rCRP purification using Ni^2+^-NTA resin (**Figure 5A**) depicts both the flow-through (lane 8) and elution stages (lanes 1-7). Analysis of the elution fraction (lanes 1-7) revealed a distinct rCRP band appeared at approximately 30 kDa, while the flow-through fraction (lane 8) exhibited an absence of the rCRP band. However, some impurities with molecular weights of approximately 60 and 180 kDa may arise from residual proteins present in cell debris. Negative staining electron microscopy revealed that rCRP, purified through a Ni^2+^ NTA resin column, exhibited an intact pentameric structure, albeit with the presence of residual miscellaneous and heterogeneous proteins (**Figure 5B**). Consequently, additional purification procedures are necessary to eliminate impurities from the elution fraction.

The SDS-PAGE analysis indicates a substantial enrichment of rCRP after elution with imidazole, particularly when applying the medium portion to a Ni^2+^-NTA column (**Figure 5B**). Further purification was accomplished through a Pierce™ p-Aminophenyl Phosphoryl Choline Agarose Fast Flow column, which exploits the binding affinity of pentameric CRP for phosphorylcholine (PCh) (**Figures 5C**, **D**). The pentameric structure of the purified rCRP is evident, as other forms of rCRP would not bind to the column. Additionally, misfolded proteins, which may occur due to incorrect disulfide bond formation, are not expected to bind correctly (9).

The heterologous expression of rCRP benefits from the eukaryotic environment, which mitigates the aggregation and protein misfolding typical in bacterial systems (9, 39). The conservative nature of chaperone proteins, coupled with similarities in cytoplasmic and organelle composition, could explain this observation. Furthermore, the use of the *S. cerevisiae* α-factor prepropeptide signal sequence has been shown to improve extracellular secretion efficiency (48). After cell removal from the medium, a two-step purification process involving Nickel Sepharose beads and p-aminophenylphosphorylcholine columns is employed to purify the secreted proteins from the supernatant. Coomassie-stained protein gels revealed reduced contamination by other proteins when the rCRP-6×His fusion protein was purified using both columns (**Figure 5**). Any interaction or purification of CRP with PCh or PCh-coated particles (PCh-conjugated agarose column) is restricted to the intact CRP homopentamer (**Figures 5C, E**). The Western blot results demonstrated the specific antibody’s recognition of rCRP produced by yeast (**Figure 5F**). Subsequent mass spectrometry analysis identified two peptides that matched with rCRP, confirming the appropriateness of its sequence (**Figure 5G** and **Table 1**).

**Table 1:** Displays the peptide segments identified through mass spectrometry analysis of rCRP.

Sequence	m/z	MH+ [Da]	AA positions
SFSIFSYATK	575.80	1150.59	46-55
VFSPNVLNWR	616.34	1231.67	177-186

*P. pastoris* shows promise as the favored host for expressing rCRP protein due to its manageable nature and posttranslational modification patterns akin to mammalian cells. Through this novel expression system, a soluble CRP-6×His fusion protein was effectively produced and released into the culture medium. While the purification of endogenous rCRP from rat blood under physiological conditions is achievable, it is complicated by the presence of SAP contamination. Recombinant rCRP was efficiently produced in *E. coli* (BL21) and *P. pastoris* GS115, and subsequently secreted into the extracellular environment. In the *E. coli* expression system, despite adding the signal peptide ALP to the N-terminus of rCRP, only a small portion of rCRP was secreted extracellularly, with the majority forming inclusion bodies *in vivo*. This issue might be due to the lack of posttranslational modifications or inadequate conditions for facilitating disulfide bond formation, necessary for proper protein folding. The use of detergents or chaperones can assist in protein solubilization or folding processes. Each subunit of rCRP contains four cysteine residues (C36, C95, C208, and C209) that are involved in forming intrachain or interchain disulfide bonds (18, 21). The conservative subunit disulfide bond between C36 and C95 is observed in human CRP, along with an additional disulfide bond between C36 and C97. Furthermore, in the pentameric structure of rCRP, the subunits C208 and C209 appear to form disulfide bonds with three other subunits. Specifically, a dimerization occurs between two monomers through a disulfide bond involving C-208 and C-209. These same cysteine sites participate in intramolecular disulfide bonds within the remaining three monomers.

## Conclusion

In this investigation, rCRP was effectively expressed and purified utilizing *E. coli* and *P. pastoris* expression platforms. Findings indicated the superiority of the *P. pastoris* GS115 (Mut^+^) strain over *E. coli* (BL21) in producing recombinant proteins, attributed mainly to its capability to execute post-translational modifications mirroring those of mammalian cells. This characteristic is essential for the correct folding and functioning of rCRP, which harbors multiple crucial disulfide bonds for its pentameric structure. The purification procedure comprised a two-step batch binding technique utilizing Nickel Sepharose beads and Pierce™ p-Aminophenyl Phosphoryl Choline Agarose resin, resulting in the isolation of highly pure rCRP. The purity and integrity of recombinant rCRP were confirmed through SDS-PAGE and electron microscopy analyses. The prokaryotic system demonstrated higher levels of impurities and lower yields in rCRP production compared to the eukaryotic system. The *P. pastoris* system offered a more conducive environment for rCRP expression and secretion, establishing it as a superior host for rCRP production with enhanced efficiency and effectiveness. This optimized approach improves both the yield and purity of rCRP, facilitating its application in structural and functional studies.

In conclusion, the *P. pastoris* GS115 Mut^+^ strain, characterized by its methanol utilization plus phenotype, exhibits superior recombinant protein production capabilities compared to the *E. coli* BL21 strain. Given its user-friendly nature and posttranslational modification profiles akin to mammalian cells, *P. pastoris* emerges as a promising candidate for expressing rCRP proteins. The soluble CRP-6×His fusion protein was effectively produced and released into the culture medium using this novel expression system. The purification of the 6×His-tagged rCRP fusion protein involved a two-step process utilizing Nickel Sepharose beads and Pierce™ p-Aminophenyl Phosphoryl Choline Agarose resin, resulting in the isolation of a protein of high purity.

## Data Availability

The original contributions presented in the study are included in the article/supplementary material. Further inquiries can be directed to the corresponding author.
